# Collagen IV^α345^ dysfunction in glomerular basement membrane diseases. III. A functional framework for α345 hexamer assembly

**DOI:** 10.1016/j.jbc.2021.100592

**Published:** 2021-03-26

**Authors:** Vadim Pedchenko, Sergei P. Boudko, Mary Barber, Tatiana Mikhailova, Juan Saus, Jean-Christophe Harmange, Billy G. Hudson

**Affiliations:** 1 Department of Medicine, Division of Nephrology and Hypertension, Vanderbilt University Medical Center, Nashville, Tennessee, USA; 2 Center for Matrix Biology, Vanderbilt University Medical Center, Nashville, Tennessee, USA; 3 Department of Biochemistry, Center for Structural Biology, Vanderbilt University, Nashville, Tennessee, USA; 4 Aspirnaut, Vanderbilt University Medical Center, Nashville, Tennessee, USA; 5 Department de Bioquímica y Biología Molecular at Facultad de Medicina y Odontología, University de València, Valencia, Spain; 6 Goldfinch Bio, Cambridge, Massachusetts, USA; 7 Department of Pathology, Microbiology and Immunology, Vanderbilt University Medical Center, Nashville, Tennessee, USA; 8 Center for Structural Biology, Vanderbilt University, Nashville, Tennessee, USA; 9 Department of Cell and Developmental Biology, Vanderbilt University, Nashville, Tennessee, USA; 10 Vanderbilt-Ingram Cancer Center, Vanderbilt University, Nashville, Tennessee, USA; 11 Vanderbilt Institute of Chemical Biology, Vanderbilt University, Nashville, Tennessee, USA

**Keywords:** AFM, atomic force microscopy, AS, Alport syndrome, DN, diabetic nephropathy, GBM, glomerular basement membrane, GP, Goodpasture’s disease, LCL, loop-crevice-loop, SEC, size-exclusion chromatography, TBS, tris-buffered saline

## Abstract

We identified a genetic variant, an 8-residue appendage, of the α345 hexamer of collagen IV present in patients with glomerular basement membrane diseases, Goodpasture’s disease and Alport syndrome, and determined the long-awaited crystal structure of the hexamer. We sought to elucidate how variants cause glomerular basement membrane disease by exploring the mechanism of the hexamer assembly. Chloride ions induced *in vitro* hexamer assembly in a composition-specific manner in the presence of equimolar concentrations of α3, α4, and α5 NC1 monomers. Chloride ions, together with sulfilimine crosslinks, stabilized the assembled hexamer. Furthermore, the chloride ion–dependent assembly revealed the conformational plasticity of the loop-crevice-loop bioactive sites, a critical property underlying bioactivity and pathogenesis. We explored the native mechanism by expressing recombinant α345 miniprotomers in the cell culture and characterizing the expressed proteins. Our findings revealed NC1-directed trimerization, forming protomers inside the cell; hexamerization, forming scaffolds outside the cell; and a Cl gradient–signaled hexamerization. This assembly detail, along with a crystal structure, provides a framework for understanding hexamer dysfunction. Restoration of the native conformation of bioactive sites and α345 hexamer replacement are prospective approaches to therapeutic intervention.

Prominent diseases of the glomerular basement membrane (GBM), a specialized form of extracellular matrix, are diabetic nephropathy (DN), Alport syndrome (AS), and Goodpasture’s disease (GP) (1, 2, 3). The morphological abnormalities in the GBM, ranging from thickening in DN to multilamellations in AS, and ruptures due to specific GBM attack by antibodies in GP involve structural alterations in collagen IV (2, 4, 5, 6, 7, 8, 9, 10), the major GBM component. The mechanisms whereby collagen IV enables normal GBM function or causes GBM abnormalities and dysfunction in disease are unknown. The understanding of GBM diseases requires knowledge of the pathobiology of the collagen IV^**α345**^ scaffold in relation to structure and assembly.

In Pokidysheva *et al.* (11), we found that the α345 hexamer, a key connection module within the of the collagen IV^α345^ scaffold, is a focal point of bioactivity, enabling GBM function. In Boudko *et al.* (12), we solved the crystal structure of the collagen IV^α345^ hexamer, which revealed the loop-crevice-loop (LCL) bioactive sites that include GP hypoepitope loops. The crystal structure also revealed a ring of chloride ions at the trimer–trimer interface, which may signal hexamer assembly analogous to collagen IV^**α112**^ hexamer (13, 14). In α345 hexamer, chloride ions may play an additional role in the conformational stability of the LCL bioactive sites, for which perturbations can lead to GP and AS. Here, we sought to explore these chloride roles in the collagen IV^**α345**^ hexamer.

## Results

### Chloride ions play key role in the assembly of the α345 hexamer, a critical step in the formation of the collagen IV^α345^ scaffold

The presence of chloride ions at the trimer–trimer interface of the α345 hexamer, as described in Boudko *et al.* (12), posited a key role of the chloride ring in hexamer assembly, based on our previous finding of a signaling role of chloride ions in the assembly of the α121 hexamer (13, 14). Thus, we investigated the role of chloride in α345 hexamer assembly, using recombinant human NC1 monomers, single-chain trimers, and miniprotomers.

#### Chloride ions initiate formation and, together with sulfilimine crosslinks, stabilize the quaternary structure of the α345 hexamer

**Figure 1:**
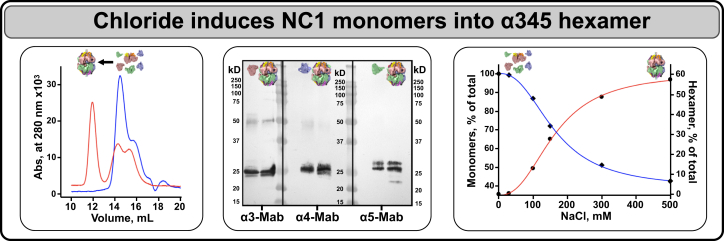
**Chloride ions signal the assembly of α345 hexamer.** Chloride ions are required for α345 hexamer assembly from recombinant NC1 monomers. Size-exclusion chromatograms of equimolar mixture of NC1 monomers in chloride-free buffer (*blue line*) and of the same mixture after incubation for 24 h at 37^o^C in the presence of chloride (*red line*), which results in formation of a hexamer peak at 12 ml and concomitant decrease of the NC1 monomer peak at 15 ml (*left* panel). Composition of the hexamer peak was established by Western blot with α3 NC1–, α4 NC1–, and α5 NC1–specific mAbs. Individual recombinant monomers were loaded in adjacent lanes as a positive control. Proteins loaded are depicted by colored pictograms at the *top* (*middle* panel). Dose dependency of the chloride effect on the assembly of α345 hexamer (*red line*) and concomitant decrease of NC1 monomers (*blue line*) with increasing concentration of Cl^-^ (*right panel*).

**Figure 2:**
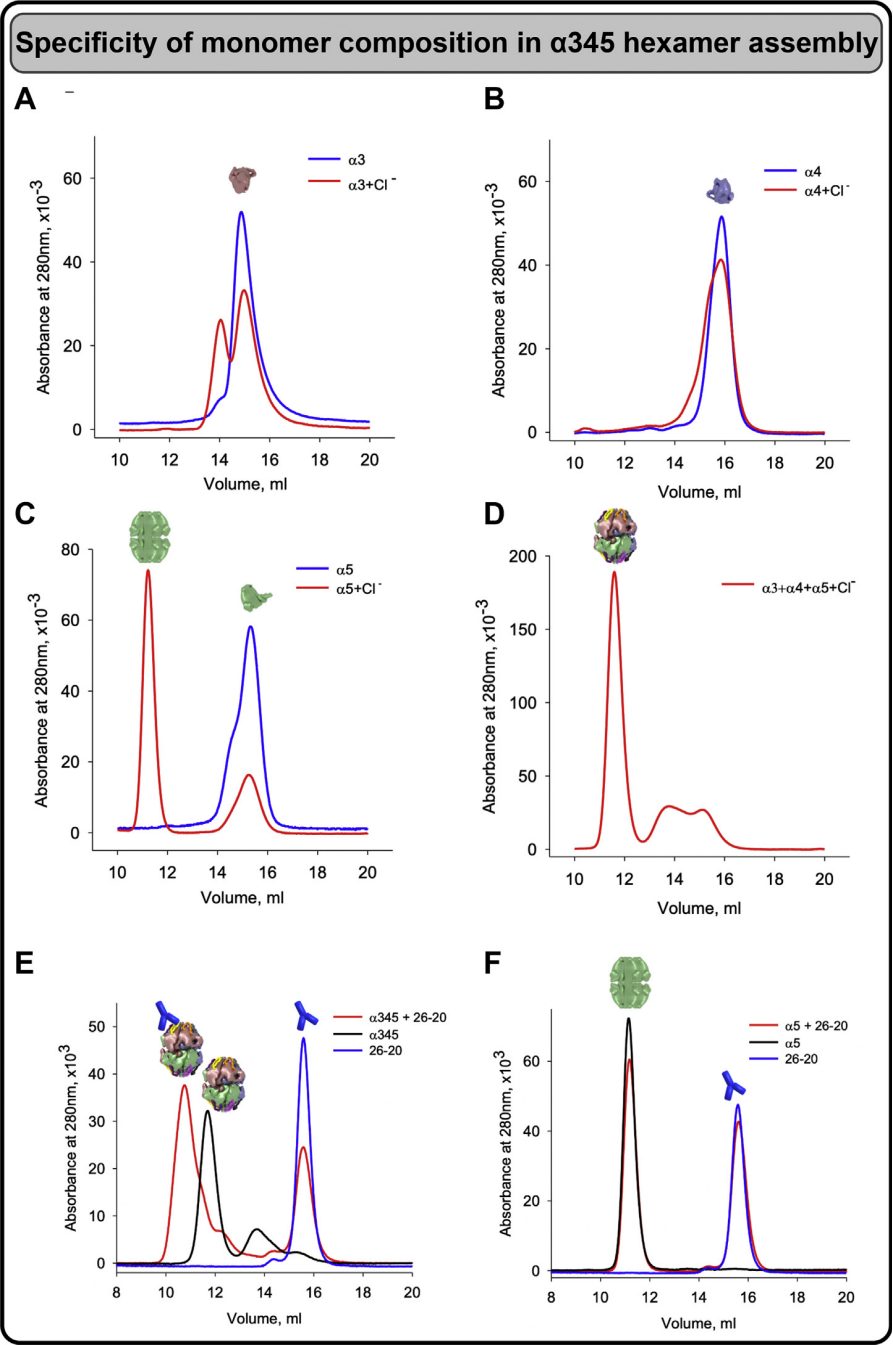
**Specificity of monomer composition in chloride induced α345 hexamer assembly.** Chloride induces assembly of the homohexamer from human recombinant α5NC1 (*C*), but not α3NC1 (*A*) or α4NC1 (*B*) monomers as demonstrated by SEC after preincubation of monomers in the presence of 150-mM NaCl (*red lines*). *Blue lines* show elution profiles of the original NC1 monomers monitored by absorbance at 280 nm. *D*, assembly of the α345 heterohexamer from the equimolar mixture of α3, α4, and α5 NC1 monomers in the presence of chloride as a positive control. Color-coded pictograms above elution profiles show position of the hexamer and monomer peaks. *E*, heterohexamer assembled in the presence of chloride from the equimolar mix of recombinant α3, α4, and α5 NC1 monomers binds the fragment antigen-binding (Fab) fragment of monoclonal antibody (Mab) 26-20 as indicated by the disappearance of hexamer peak at 12 ml concomitant with the formation of the hexamer–Fab complex peak at 10.5 ml and reduction of the free Fab peak at 15.6 ml. *F*, in contrast, 26-20 Fab does not bind to isolated α5NC1 homohexamer as indicated by the absence of changes in the position and areas of the hexamer or Fab peaks. SEC, size-exclusion chromatography.

Recombinant human α3, α4, and α5 NC1 monomers were incubated with 150-mM NaCl at 37 °C for 24 h. Separation by size-exclusion chromatography (SEC) revealed a new hexamer peak and concomitant decrease of the NC1 monomer peak (Fig. 1, *left*). The hexamer peak contained α3, α4, and α5 NC1 monomers (Fig. 1, *middle*) and its intensity directly correlated with the concentration of chloride ions (Fig. 1, *right*). Additional studies revealed that specificity for assembly is encoded in the monomer subunits, which, in the presence of chloride, interact to form an α345 hexamer (Fig. 2, *A*–*D*). This heterohexamer was a predominant species formed in the presence of all three NC1 monomers (Fig. 2*E*), whereas α5NC1 homohexamer was essentially absent (Fig. 2*F*).

**Figure 3:**
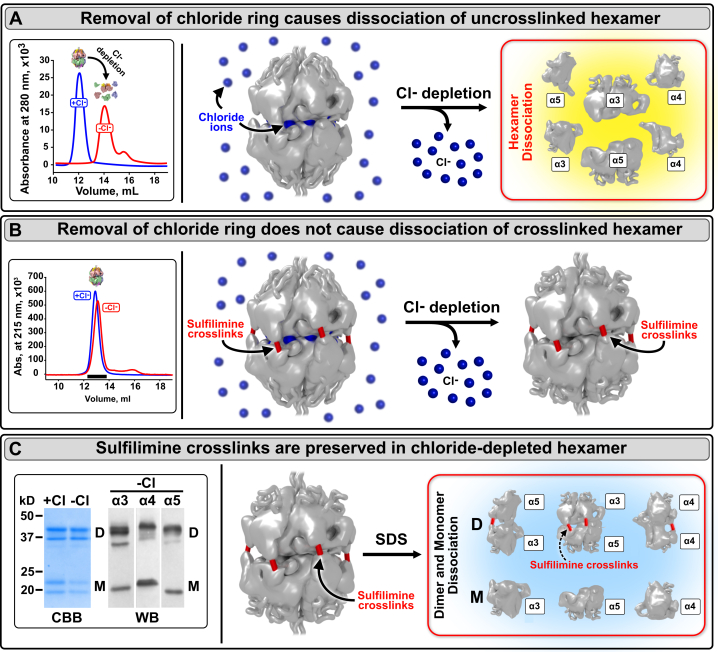
**Chloride ring stabilizes α345 hexamer, and sulfilimine crosslinks reinforce hexamer structure.***A*, depletion of chloride caused dissociation of uncrosslinked α345 hexamers as demonstrated by size-exclusion chromatography. Elution profiles in either the presence of 150-mM NaCl or in chloride-free buffer are shown in *blue* and *red* as indicated in the *left side*. *B*, the removal of chloride ions did not cause dissociation of cross-linked hexamers as demonstrated by size-exclusion chromatography. Elution profiles of human native GBM hexamers in the presence and in the absence of Cl^-^ are shown in *blue* and *red* as indicated in the *left side*. The *black bar* under the elution profile indicates the expected elution volume of the NC1 hexamer. *C*, chloride-depleted hexamers, shown in *panel B* (*far right),* were subjected to SDS-PAGE revealing prominent dimer bands (*left side*), indicating sulfilimine crosslinks were not affected (*right side*).

Once assembled, chloride ions stabilize the hexamer structure, as revealed by the dissociation of uncrosslinked hexamer into subunits upon removal of chloride ions (Fig. 3*A*). In contrast, removal of chloride does not induce dissociation of a crosslinked hexamer, revealing that sulfilimine crosslinks (15) reinforce the hexamer for stability (Fig. 3, *B* and *C*).

**Figure 4:**
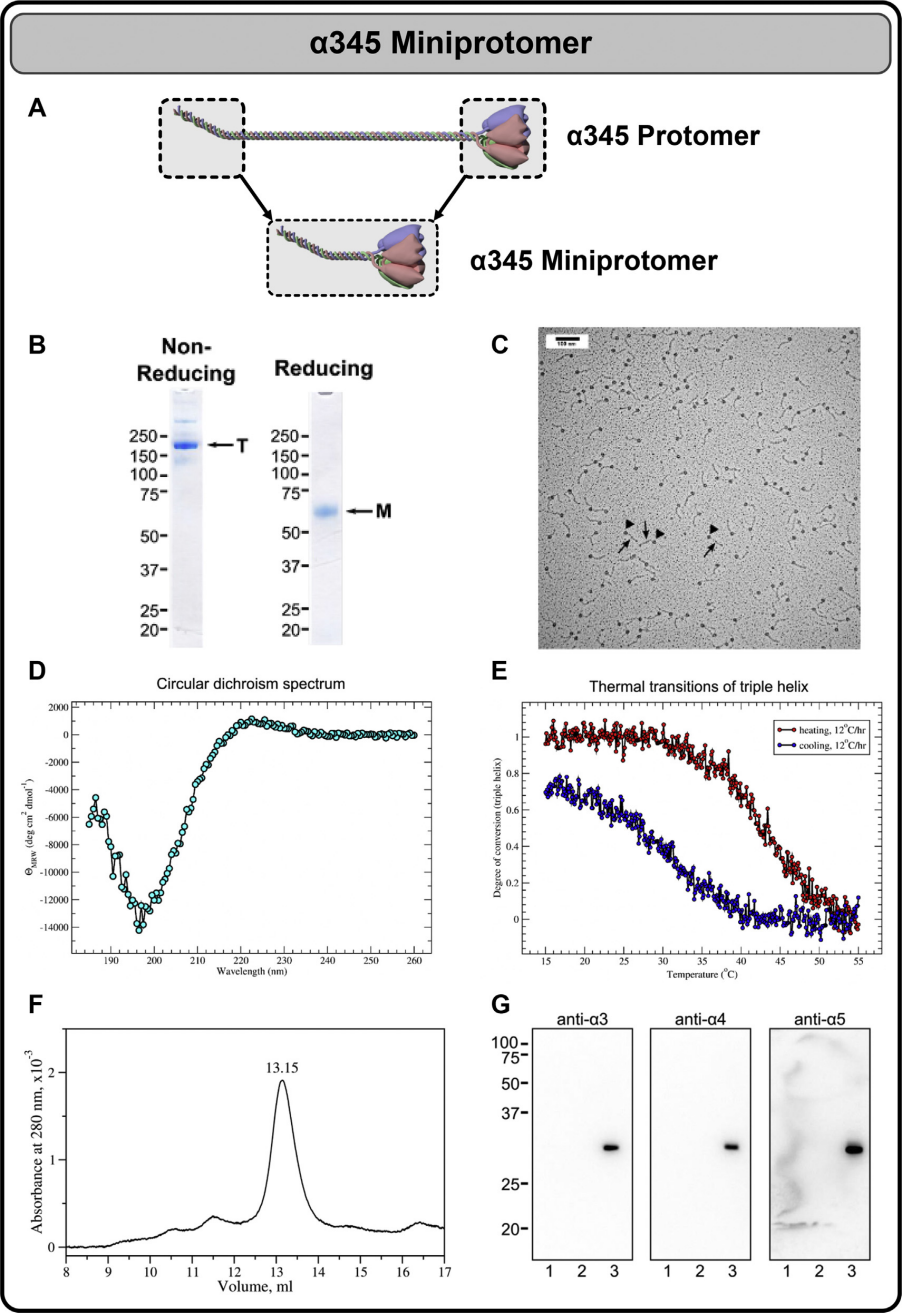
**Production and characterization of a recombinant α345 miniprotomer.***A*, the α345 miniprotomer was engineered and expressed in mammalian cells and is schematically shown in comparison to a full-length α345 protomer. *B*, purified α345 miniprotomer after binding and elution from anti-FLAG M2-agrose. SDS-PAGE under nonreducing and reducing conditions revealed formation of the trimer stabilized by interchain disulfide bonds. Bands corresponding to monomer (M) and trimer (T) are indicated. *C*, rotary shadowing of the α345 miniprotomer. The rotary shadowing electron microscopy data showing individual ∼70-nm-long α345 miniprotomers each containing a triple helical collagenous domain (*arrows*) with a globular NC1 trimer (*triangles*) at the end. *D*, CD spectrum of the α345 miniprotomer in 20-mM sodium phosphate buffer, pH 6.5, at 15 °C. *E*, thermal unfolding upon heating and partial refolding upon cooling of the triple helical part of the miniprotomer. The apparent melting temperature is 43 °C at the heating rate of 0.2 °C/min. Hysteresis observed between heating and cooling transitions is a characteristic feature for collagen triple helix transitions (26). *F*, size-exclusion chromatography of α345 miniprotomer digested with collagenase. The column was equilibrated and operated in tris-buffered saline (TBS) buffer containing 150-mM Cl^-^. The position of a major peak at 13.15 ml corresponds to the NC1 hexamer. *G*, Western blot of the major peak (lane 3, ∼10 ng/lane) using chain-specific antibodies against α3, α4, and α5 NC1. Recombinant murine α1 and α2 NC1 domains (50 ng/lane) were loaded as negative controls in lanes 1 and 2.

**Figure 5:**
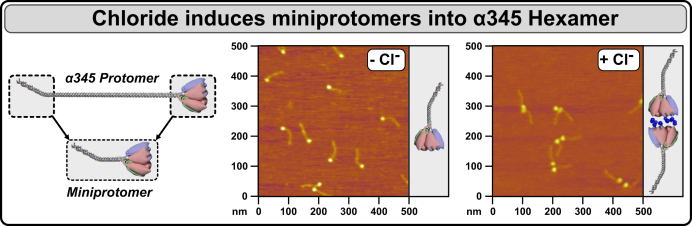
**Chloride ions signal the assembly of and stabilize α345 miniprotomers.** The α345 miniprotomer was engineered and expressed in mammalian cells (*left*). Analysis of α345 miniprotomers by atomic force microscopy (AFM) in the chloride-free buffer revealed the presence of individual miniprotomers (*middle*). In the presence of chloride, head-to-head association of two miniprotomers was observed (*right*), suggesting chloride-mediated miniprotomer dimerization *via* NC1 domain, thus confirming functionality of α345 miniprotomers and demonstrating their potential to incorporate into collagen IV^α345^ scaffold in tissues.

We sought to validate these *in vitro* results in a biological system. To this end, we expressed and characterized a recombinant α345 miniprotomer in CHO cells in culture. The purified miniprotomer was confirmed to be composed of the α345 chains (Fig. 4). Rotary shadowing and atomic force microscopy (AFM) revealed individual molecules, characterized as ∼70-nm-long miniprotomers containing the triple helical collagenous domain with the globular NC1 trimer at the end (Figs. 4*C* and 5). In the presence of chloride, the miniprotomers associated head-to-head (Fig. 5), indicating that chloride ions mediate the protomer dimerization, a key step in scaffold assembly outside the cell (16) (see Supplementary Section 3 for additional data).

Collectively, these studies demonstrate that the α3, α4, and α5 NC1 monomers are encoded with structural determinants that govern both chain selection in protomer assembly and chloride-dependent oligomerizations of specific protomers into the collagen IV^α345^ scaffold in the GBM.

#### Chloride ions stabilize the conformation of the α345 hexamer and reveal conformational plasticity of the LCL sites

**Figure 6:**
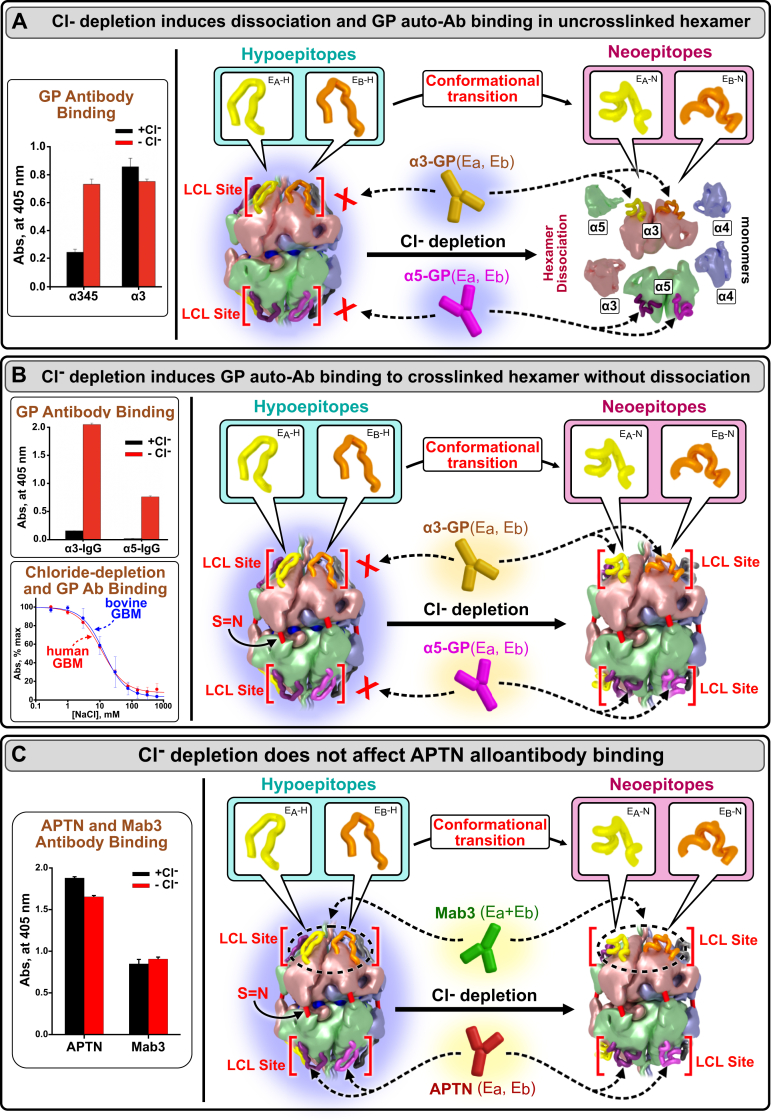
**Removal of chloride ring enables GP autoantibody binding, revealing conformational plasticity of LCL site.***A*, chloride depletion causes dissociation of uncrosslinked α345 hexamer and GP antibody binding to α3NC1 monomer. *Left* panel: While binding of the purified α3-GP autoantibody to recombinant human α345 hexamer was significantly increased in the absence of chloride, binding to the α3NC1 monomer was unaffected, suggesting that GP epitopes are fully exposed in monomer subunits. *Right* panel: the mechanism of chloride-dependent α345 hexamer dissociation and GP antibody binding. *B*, depletion of chloride causes GP antibody binding without dissociation of the native crosslinked α345 hexamers from human and bovine GBM. *Left panels:* the α345 hexamer did not bind purified α3-GP autoantibody in the presence of 150-mM chloride but robust antibody binding was observed upon incubation in the Cl-free buffer. Chloride depletion also induced binding of the GP α5 autoantibodies to the GBM hexamer similar to purified α3 autoantibodies, suggesting general chloride-dependent mechanism for the E_A_ and E_B_ epitope exposure in both the α3 and α5 NC1 subunits of the hexamer (*top left)*. The extent of GP autoantibody binding to GP autoantigen from bovine or human glomerular basement membrane was dependent upon the Cl^-^ concentration (*bottom left*). *Right panel*: the overall mechanism of chloride-dependent GP antibody binding to α345 hexamer. In the presence of sulfilimine crosslinks (S = N), depletion of chloride ions induces conformational change within E_A_ and E_B_ loops without hexamer dissociation. This conformational change enables transition from the hypoepitope to the neoepitope loop configuration, thus enabling binding of the GP autoantibody. *C*, binding of human Alport post-transplant nephritis patient (APTN) antibody to the epitope located within the α5 LCL site is independent of chloride (data—*left panel;* cartoon representation—*right panel).* Similarly, Mab3 mAb binding to E_A_ and E_B_ epitopes within the α3 LCL site is also independent of chloride. These results demonstrate that GP autoantibodies and APTN alloantibody binding require distinct conformations, thus revealing conformational plasticity of the α3 and α5 LCL sites. GP, Goodpasture’s disease; GBM, glomerular basement membrane.

Based on our previous findings, a conformational transition occurs upon dissociations of α345 hexamer with 6 M GuHCl, a strong protein denaturant. This conformational transition was revealed by GP autoantibodies, which bound only the disassociated monomer and crosslinked subunits (17, 18). Herein, we observed that, under nondenaturing conditions, the removal of chloride ions also induced GP antibody binding to both α3 and α5 subunits (Fig. 6*A*), concomitantly with the dissociation of the noncrosslinked hexamer (Fig. 3*A*). Moreover, removal of chloride ions also induced antibody binding to the α3 and α5 subunits within a cross-linked hexamer (Fig. 6*B*), even without hexamer dissociation (Fig. 3*B*). There was a dose-dependent increase in binding of α3-GP autoantibody up to 20-fold upon decreasing of the chloride concentration (Figs. 6*B* and S5).

In contrast, chloride removal did not impact the binding of human Alport post-transplant anti-GBM nephritis alloantibody to the α5 subunit of the crosslinked hexamer (Fig. 6*C*, *left*). Our previous work has established that the epitope for Alport post-transplant anti-GBM nephritis alloantibody and hypoepitope for the GP autoantibody have the same primary structures but differ in their conformations based on hexamer dissociation using strong denaturants (19, 20, 21). This conformational distinction was now verified under nondenaturing conditions by the differential effect of chloride on binding of these two antibodies to the crosslinked hexamer. Furthermore, chloride removal also did not affect the binding of mouse mAb Mab3 (Fig. 6*C*). This antibody targets the α3 GP hypoepitopes in the intact hexamer and the GP neoepitopes in the dissociated hexamer and can block GP autoantibody binding (22). Thus, only a few residues may account for a critical conformational transition from hypoepitope to neoepitope required for GP autoantibody binding within the LCL site.

## Discussion

**Figure 7:**
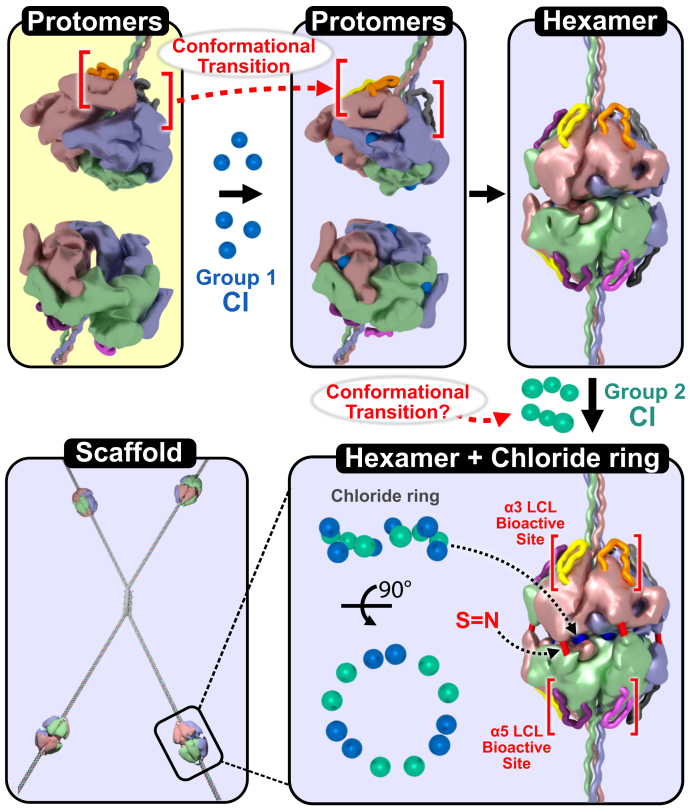
**Chloride ions signal assembly of, and together with sulfilimine crosslinks, stabilize the α345 hexamer of the collagen IV scaffold.** The overall mechanism of α345 hexamer and scaffold assembly. The assembly of two α345 protomers into a α345 hexamer requires consecutive binding of two groups of chloride ions forming a 12-ion chloride ring. The X-ray structure of α345 hexamer revealed that chloride ions of group 1 are buried within the inner cavities, whereas the chloride ions of group 2 are located in more shallow pockets. Chloride ions initiate protomer oligomerization and stabilize the hexamer structure, which includes a conformational transition in the LCL bioactive site, forming the collagen IV^α345^ scaffold. Furthermore, sulfilimine crosslinks (S = N) reinforce hexamer stability.

Collectively, our findings reveal details of the assembly mechanisms of the collagen IV^α345^ hexamer. These include NC1-directed oligomerization, chloride signaling and stabilization of conformation, and sulfilimine crosslink reinforcement as summarized in Figure 7. Furthermore, a dynamic feature of conformational plasticity of the LCL bioactive site on the surface of the α345 hexamer was demonstrated (Fig. 7). This assembly detail, along with the crystal structure (12), provides a framework for understanding how genetic variants cause hexamer dysfunction and for the development of therapy. The variants could interfere with multiple assembly steps, including the formation and conformation of the LCL bioactive sites. This conformation can be perturbed by the Z-appendage and other genetic variants that occur in the hexamer in AS, endogenous and exogenous triggers in GP, and hyperglycemia in DN (11, 12). The restoration of the native conformation of these sites and/or replacement of the hexamer are prospective approaches to therapeutic intervention.

## Experimental procedures

### Design, expression, and purification of α345 miniprotomers

Codon-optimized synthetic DNA–encoding collagen IV α3, α4, and α5 minichains extended with chain-specific tags were cloned into proprietary vectors and used for transient coexpression in ExpiCHO-S cells (performed at Aragen Bioscience, Inc). Secreted proteins were purified on a Ni column, and α345 miniprotomer was purified using anti-FLAG M2-Agarose (Sigma). The chain composition was confirmed by Western blotting using NC1 chain-specific antibodies H3 (anti-α3), H4 (anti-α4), and Mab5 (anti-α5).

### Expression and purification of α3, α4, and α5 NC1 monomers

Recombinant human NC1 domains were amplified by PCR from human kidney cDNA library, cloned in the derivative of pRc-CMV mammalian expression vector that includes BM-40 signal peptide and N-terminal FLAG tag, and transfected into HEK-293 cells using Hepes-calcium phosphate (ProFection, Promega). Stable clones were selected using neomycin (0.4 mg/ml), and clones with the highest levels of NC1 expression after testing by Western blotting were expanded into T225 culture flasks. The conditioned medium was collected from confluent cultures two times a week, and recombinant proteins were purified by passing through anti-FLAG M2-agarose (Sigma) columns with subsequent elution with FLAG peptide (100 μg/ml, Sigma) and concentration on ultrafiltration concentrators (Amicon 10MWCO, Millipore) to 2 to 4 mg/ml. Proteins were further purified by SEC on Superdex 200 column in the TBS buffer (23).

### Rotary shadowing electron microscopy

The sample was dialyzed against a volatile buffer, 50-mM ammonium bicarbonate, pH 6.8. The concentration was adjusted to 100 μg/ml. 30 μl of the sample was mixed with 70 μl of pure glycerol and sprayed onto a freshly cleaved mica surface as ∼50 μm drops. The mica was then placed into a vacuum evaporator and dried under vacuum. Contrast was affected by evaporating Pt-C at a 6^o^ angle relative to the mica surface as the sample rotated. Further processing and electron microscope imaging was performed as described (24).

### AFM

The sample preparation for AFM was performed on mica (Highest Grade V1 AFM Mica Discs, 10 mm, Ted Pella). The protein samples were diluted to a 1 to 2 μg/ml, and 50 μl was deposited onto freshly cleaved mica. After a 30-s incubation, the excess of unbound proteins was washed off with the ultrapure water for ∼10 s, and the mica was immediately dried under filtered air. All proteins were imaged under dry conditions, and the solution conditions of the samples refer to the conditions in which they were deposited onto mica. AFM imaging was performed on Asylum Research MFP-3D atomic force microscope using the AC tapping mode in air. AFM tips with a 160-kHz resonance frequency and 5 N/m force constant (MikroMasch, HQ: NSC14/AL BS) were used.

### CD spectroscopy

CD spectra were recorded on a Jasco model J-810 spectrometer equipped with a Peltier temperature control unit (JASCO Corp.) using a quartz cell of 1-mm path length at 15 °C. The spectra were normalized for the concentration and path length to obtain the mean molar residue ellipticity. Thermal scanning curves were recorded at 225 nm with the heating rate of 0.2 °C/min.

### SEC

SEC of tissue-extracted and recombinant NC1 hexamers was conducted with a Superdex 200 Increase 10/300GL gel filtration column (GE Healthcare), using an ÄKTA purifier (GE Healthcare) at a flow rate of 0.5 ml/min using 25-mM Tris HCl, pH 7.5, 150-mM NaCl (TBS, +Cl^−^ buffer), 25-mM Tris acetic acid, pH 7.5, or 150-mM sodium acetate (Cl^−^-free buffer). Eluting proteins were monitored by absorbance at 280 nm. Apparent sizes were calculated using a calibration curve where logarithm of the molecular mass of protein standards (Bio-Rad) was plotted against normalized retention volume (25). The area of hexamer peak was integrated using Unicorn software (GE Healthcare) and expressed as a percentage of the total peak area for quantitation of hexamer assembly.

### α345 hexamer assembly

*In vitro* assembly of the recombinant NC1 monomers and trimers was initiated by the addition of NaCl to concentrated proteins (2 mg/ml) in 25-mM Tris acetic acid buffer, pH 7.5, followed by incubation for 24 h at 37 °C. The products of reaction were fractionated and analyzed by SEC FPLC in TBS as described above.

## Data availability

All data described in this article are available in the main text or supporting information. The atomic coordinates and structure factors (code 6wku) have been deposited in the Protein Data Bank (http://www.pdb.org/).

## Supporting information

This article contains supporting information.

## Conflict of interest

The authors declare that they have no conflicts of interest with the contents of this article.
