# Supplementary material for: Collagen IVα345 dysfunction in glomerular basement membrane diseases. III. A functional framework for α345 hexamer assembly
Source: J Biol Chem. 2021 Mar 26;296:100592. doi: 10.1016/j.jbc.2021.100592 (PMC8099640; doi:10.1016/j.jbc.2021.100592)
Supplement: Supplementary Material [file mmc1.docx]

**SUPPLEMENTARY APPENDIX**

This appendix has been provided by the authors to give readers additional information about their work.

Supplement to: Mechanisms of Collagen IV345 assembly and dysfunction in Goodpasture’s and Alport diseases: III. Chloride ions signal assembly and stabilize conformation.

**Table of Contents**

|  | Page | |
| --- | --- | --- |
| **Supplementary Section 1: *Role of chloride ions in initiation of α345 hexamer assembly*** | | 2 |
| **Supplementary Section 2: *Role of chloride ions in stabilization of α345 hexamer structure*** *(Supplementary Fig. 1)* | | 2 |
| **Supplementary Section 3: *Verification of the role of chloride in the cell system using α345 collagen IV miniprotomer*** (*Supplementary Figs. 2-4*) | | 4 |
| **Supplementary Section 4: *Role of chloride ions in Goodpasture’s antibody reactivity with α345 hexamer of collagen IV*** (*Supplementary Fig. 5*) | | 8 |

**Supplementary Section 1: Role of chloride ions in initiation of α345 hexamer assembly**

We investigated the role of chloride ions in collagen IV α345 hexamer assembly using recombinant human NC1 monomers. This strategy provides ultimate flexibility in analyzing the specificity and kinetics of hexamer assembly under controlled conditions *in vitro* and has been employed for the mechanism of assembly of α121 collagen hexamer (1,2). Recombinant human α3, α4 and α5 NC1 monomers were isolated from conditioned medium of HEK 293 cells using affinity and size-exclusion chromatography. The monomers were concentrated in chloride-free buffer, combined at equimolar ratio, and incubated with 150 mM NaCl at 37**°**C for 24h. After separation by SEC in TBS, this results in formation of a new peak with higher molecular weight (Main Text Fig. 1A, *left*) and concomitant decrease of NC1 monomers peak eluted at 15 ml. We identified this novel peak as a hexamer based on the elution profile of the control NC1 hexamer isolated from human GBM. Western blot with chain-specific monoclonal antibodies demonstrated the presence of both α3, α4 and α5 NC1 monomers in this peak (Main Text Fig. 1A, *middle*). Furthermore, we showed that effect of Cl^-^ on α345 hexamer assembly is dose-dependent and concomitant with a decrease in constituent NC1 monomers (Main Text Fig. 1A, *right*).

Given the recently published crystal structures of the individual α3, α4 and α5 NC1 homo-oligomers(3), we examined the possibility that observed hexamer peak might represent a mixture of the individual NC1 homohexamers. In the presence of chloride, only α5, but not α3 or α4 NC1 monomers formed homohexamer under physiologic conditions (Main Text Fig. 2), consistent with the previous findings(3). When the equimolar mixture of α3, α4 and α5 NC1 monomers was used for assembly, the major assembled form was the α345 NC1 heterohexamer (Main Text Fig. 1B-D).

**Supplementary Section 2: Role of chloride ions in stabilization of α345 hexamer structure**

Unlike protein denaturants or low pH treatment, which result in dissociation into dimer and monomer subunits(4) (5), purified GBM hexamer in the chloride-free buffer retained its hexameric configuration as demonstrated by the large proportion of dimers using SDS-PAGE for abundant α1/α2 NC1 and Western blot for α3, α4 and α5 NC1 subunits (Supplementary Fig. 1). This is in contrast with the hexamer from lens basement membrane (LBM) composed mainly from un-crosslinked α1 and α2NC1 monomers, which readily dissociates into monomers in the absence of chloride(1).

*Along with our previous data, these findings demonstrate that* *NC1 monomers contain the “code of assembly” (1,6), i.e., structural determinants that govern both chain selection in protomers and chloride-dependent assembly of the specific protomers into corresponding collagen IV^α112^ or collagen IV^α345^ scaffolds in tissues. The specific roles of NC1 domains, chloride ring, and sulfilimine crosslinks in hexamer assembly is summarized in Main Text Fig. 3.*


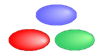

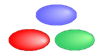

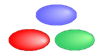

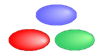

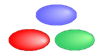

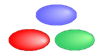

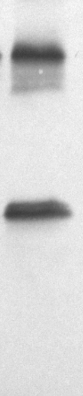

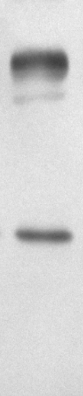

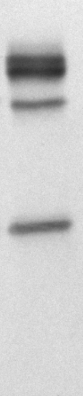


**α3**

**α4**

**α5**

**D**

**M**

**-Cl -Cl -Cl**

**D**

**M**


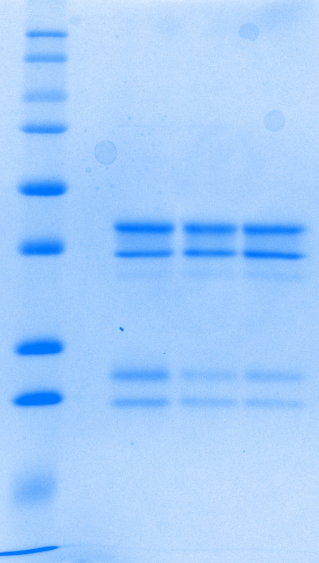


**+Cl -Cl**

Supplementary Fig. 1. Chloride depletion does not affect sulfilimine crosslink integrity in the native human GBM hexamers as evident by the relative abundance of NC1 dimers (D) compared to monomers (M) on Coomassie Blue-stained SDS-PAGE for predominant α121 hexamer (*left* panel) and Western blot for α3, α4 and α5NC1 subunits developed with NC1-specific Mabs (*right* panel). Coomassie Blue-stained SDS-PAGE for predominant α121 hexamer (*left* panel) is also used in Figure 3.

**Supplementary Section 3: Verification of the role of chloride in the cell system using α345 collagen IV miniprotomer.**

Our *in vitro* studies demonstrated that the α3, α4 and α5 NC1 monomers are encoded with structural determinants that govern both chain selection in protomer assembly and chloride-dependent oligomerizations of specific protomers into the collagen IV^α345^ scaffold in the GBM. We then sought to validate these *in vitro* results in a biological system.

To this end, we expressed and characterized a recombinant α345 mini-protomer in HEK 293 cell culture. Expression of α345 miniprotomer was achieved by cloning of the α3, α4, and α5 minichains of mouse collagen IV and by co-expressing of three proteins in suspension culture of ExpiCHO cells. Each minichain composed of authentic signal peptide, 7S domain, shortened collagenous domain and NC1 domain, thus encompassing all functional domains of the corresponding full-length α-chain with an additional advantage of smaller size for enhanced expression. Each minichain also included unique carboxy terminal tag for the affinity purification (Supplementary Fig. 2).

After purification of the culture medium using Ni-NTA column, all three minichains were detected in monomer, dimer and trimer forms by Western blot using tag-specific antibodies (Supplementary Fig. 3). Subsequent purification on anti-FLAG column in the presence of 150 mM chloride (TBS buffer) produced homogenous by SDS-PAGE protein with molecular weight corresponding to the trimer, which is stabilized by interchain disulfide bonds and was converted to monomers of ~60 kDa upon reduction (Main Text Fig. 4B). Since this protein derived from two distinct tag-affinity columns, this indicated formation of a complex between different minichains.

The rotary shadowing electron microscopy showed individual ~70 nm-long miniprotomers containing triple helical collagenous domain with globular NC1 trimer at the end (Main Text Fig. 4C). Circular dichroism spectrum of the α345 miniprotomer shows positive peak at 225 nm indicating formation of properly folded triple helical collagenous domain (Main Text Fig. 4D). Thermal unfolding indicated that collagenous domain is stable at 15-30 °C and has an apparent melting temperature of 43 °C (Main Text Fig. 4E). The observed hysteresis between heating and cooling transitions is a characteristic feature for collagen triple helix transitions(7). Digestion of the collagenous domain of α345 miniprotomer with bacterial collagenase resulted in formation of α3, α4 and α5 NC1 monomers, which were identified by SDS-PAGE and mass spectrometry (Supplementary Fig. 4). To demonstrate that monomers formed the α345 NC1 heterohexamer, we analyzed product after collagenase digest by SEC (Main Text Fig. 4F). The presence of the α3, α4 and α5 NC1 monomers in the main peak corresponding to the hexamer was established by the Western blot with specific monoclonal antibodies (Main Text Fig. 4G). *In aggregate, these data verified a technology for the recombinant production of α345 miniprotomer*.

**>mouse_a3_mini**

MDMRVPAQLLGLLLLWLRGARCKGCVCKGKGQCLCAGTKGEKGEKGVPGSPGFPGQKGFPGPEGLPGPQGPKGSPGLPGLTGPKGIRGITGLPGFAGPPGLPGLPGHPGPRGLAGLPGCNGSKGEQGFPGFPGTPGYAGLPGPDGLKGQKGEPAQGEDRGFNGKGSPGPPGVPGQPGMKGDPGPLGLPGIPGPCGPRGKPGKDGKPGTPGPAGTKGNKGLKGQQGPPGLDGLPGLKGNPGDRGTPATGTRMRGFIFTRHSQTTAIPSCPEGTQPLYSGFSLLFVQGNKRAHGQDLGTLGSCLQRFTTMPFLFCNINNVCNFASRNDYSYWLSTPALMPMDMAPISGRALEPYISRCTVCEGPAMAIAVHSQTTAIPPCPQDWVSLWKGFSFIMFTSAGSEGAGQALASPGSCLEEFRASPFIECHGRGTCNYYSNSYSFWLASLNPERMFRKPIPSTVKAGDLEKIISRCQVCMKKRHGGGGSHHHHHHHHHH*

**>mouse_a4_mini**

MDMRVPAQLLGLLLLWLRGARCKKYGGPCGGRNCSVCQCFPEKGSRGHPGPLGPQGPIGPLGPLGPIGIPGEKGERGDSGSPGPPGEKGDKGPTGVPGFPGVDGVPGHPGPPGPRGKPGVDGYNGSRGDPGYPGERGAPGPGGPPGQPGENGEKGRSVYITGGVKGLPGVPGPRGPEGAMGEPGRRGLPGPGCKGEPGPDGRRGQDGIPGSPGPPGRKGDTGEAGCPGAPGPPGPTGDPGPKGFGPGSLSGFLLVLHSQTDQEPACPVGMPRLWTGYSLLYMEGQEKAHNQDLGLAGSCLPVFSTLPFAYCNIHQVCHYAQRNDRSYWLSSAAPLPMMPLSEEEIRSYISRCAVCEAPAQAVAVHSQDQSIPPCPRTWRSLWIGYSFLMHTGAGDQGGGQALMSPGSCLEDFRAAPFVECQGRQGTCHFFANEYSFWLTTVNPDLQFASGPSPDTLKEVQAQRRKISRCQVCMKHSGGGGSDYKDDDDK*

**>mouse_a5_mini**

MDMRVPAQLLGLLLLWLRGARCAACHGCSSGSKCDCSGIKGEKGERGFPGLEGHPGLPGFPGPEGPPGPRGQKGDDGIPGPPGPKGIRGPPGLPGFPGTPGLPGMPGHDGAPGPQGIPGCNGTKGERGFPGSPGFPGLQGPPGPPGIPGMKGEPGSIIMSPLPGDPGPPGIPGQPGLKGLPGLPGPQGLPGPIGPPGDPGRNGLPGFDGAGGRKGDPGLPGQPGARGLDGPPGPDGLQGPPGPPGTTSVAHGFLITRHSQTTEAPQCPRGTVHIYEGFSLLYVQGNKRAHGQDLGTAGSCLRRFSTMPFMFCNINNVCNFASRNDYSYWLSTPEPMPMNMEPLKGQSIQPFISRCAVCEAPAVVIAVHSQTIQIPHCPQGWDSLWIGYSFMMHTSAGAEGSGQALASPGSCLEEFRSAPFIECHGRGTCNYYANSYSFWLATVDMSDMFNKPQSETLKAGDLRTRISRCQVCMKRTGGGGSYPYDVPDYA*

Supplementary Fig. 2. *Amino acid sequence of recombinant minichains of murine collagen IV α3, α4, and α5 chains.* Underlined are the signal peptide for secretion. Highlighted are His-tag (α3), FLAG-tag (α4), and HA-tag (α5).


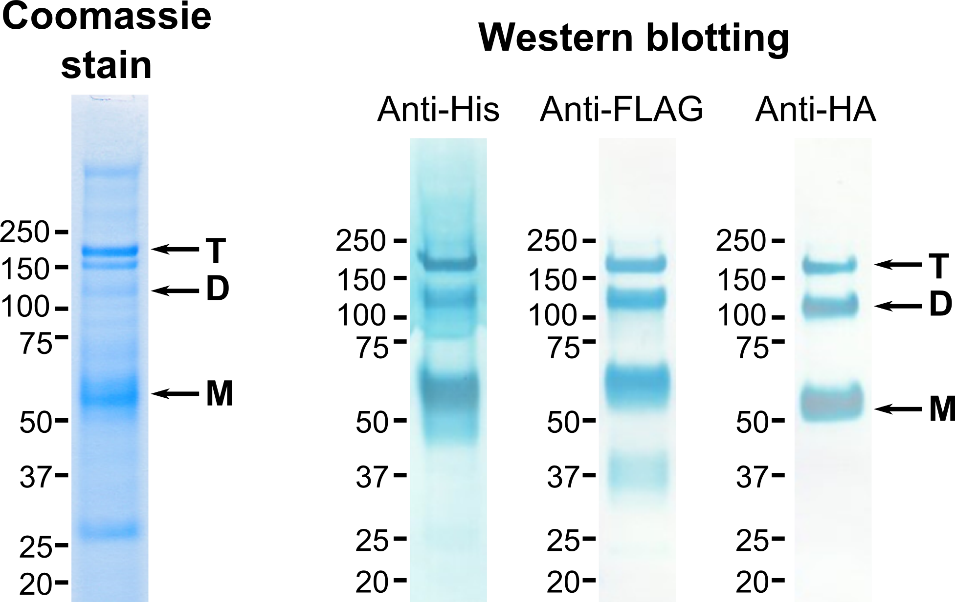


Supplementary Fig. 3. *Purification of the recombinant α345 miniprotomer*. Recombinant proteins form cell culture media were purified on Ni-NTA column for trapping of His-tagged α3-minichain and associated proteins, and analyzed by non-reducing SDS-PAGE and Western blotting with three tag-specific antibodies. Bands corresponding to minichains monomer (M), dimer (D) and trimer (T) were detected.

**
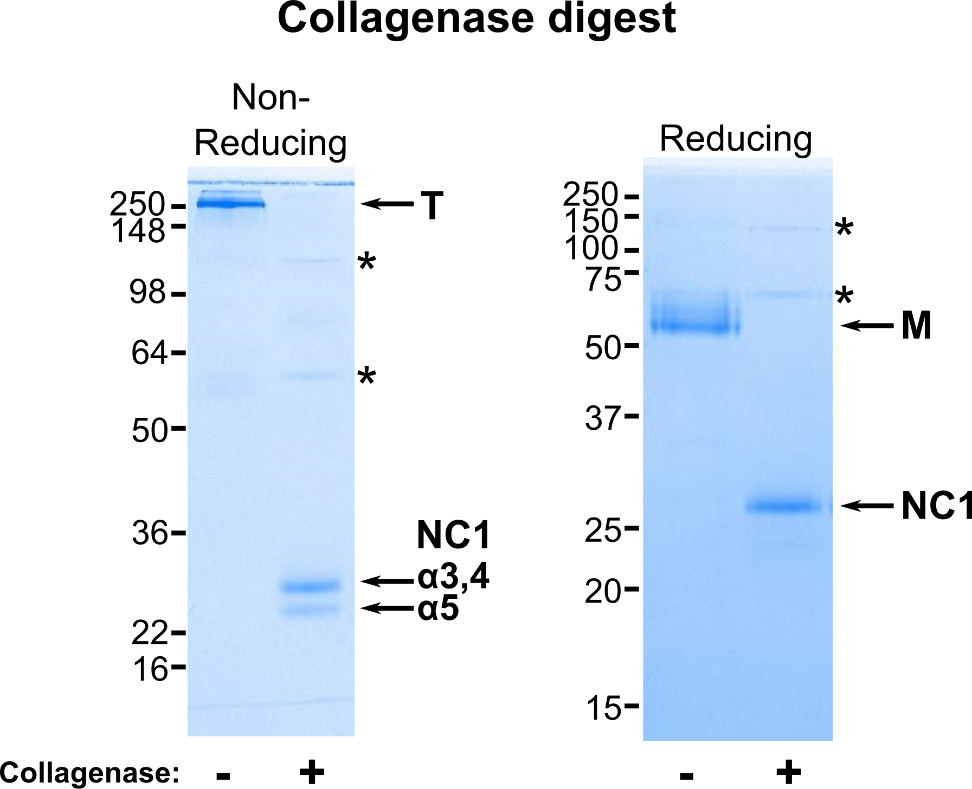
**

Supplementary Fig. 4. Collagenase digest of the α345 miniprotomer results in formation of the NC1 domains, which have been verified by mass spectroscopy. Bands corresponding to the monomer (M) and trimer (T) of the α345 miniprotomer are indicated. Bands derived from collagenase are labeled with *.

**Supplementary Section 4: Role of chloride ions in Goodpasture’s antibody reactivity with α345 hexamer of collagen IV.**

We found that GP autoantibody binding to α345 hexamer of collagen IV could be induced by depletion of chloride ions (Main Text Fig. 6A). Ablation of the chloride had similar effects on the binding of the α3-autoantibody and the α5-autoantibody, a distinct and second most abundant subset of GP autoantibodies specifically reactive to the α5NC1 domain (Main Text Fig. 6A, *left*). As we showed previously, high titers of these antibodies were associated with poor kidney outcome in GP patients (8). Furthermore, their pathogenic role was directly demonstrated by unique GP case with anti-GBM antibodies restricted to α5NC1 and in rat model of experimental autoimmune glomerulonephritis upon immunization with recombinant α5NC1(9).

To verify that this Cl^-^-dependent reactivity with GP antibodies is not an artifact due to the denaturation of the antigen on ELISA plates, we performed ELISA assay in “reversed” format by capturing of biotin-labeled GBM hexamer in solution on the plate coated with purified GP antibodies, which resulted in identical Cl^-^-dependent response, thus eliminating the possibility of denaturation of the antigen on solid phase (Supplementary Fig. 5).

Supplementary Fig. 5. Capture of the GBM hexamer labeled with biotin by purified α3-GP autoantibody coated on ELISA plate at various concentration of chloride.

**Supplementary References**

1. Cummings, C. F., Pedchenko, V., Brown, K. L., Colon, S., Rafi, M., Jones-Paris, C., Pokydeshava, E., Liu, M., Pastor-Pareja, J. C., Stothers, C., Ero-Tolliver, I. A., McCall, A. S., Vanacore, R., Bhave, G., Santoro, S., Blackwell, T. S., Zent, R., Pozzi, A., and Hudson, B. G. (2016) Extracellular chloride signals collagen IV network assembly during basement membrane formation. *The Journal of cell biology* **213**, 479-494

2. Pedchenko, V., Bauer, R., Pokidysheva, E. N., Al-Shaer, A., Forde, N. R., Fidler, A. L., Hudson, B. G., and Boudko, S. P. (2019) A chloride ring is an ancient evolutionary innovation mediating the assembly of the collagen IV scaffold of basement membranes. *J Biol Chem* **294**, 7968-7981

3. Casino, P., Gozalbo-Rovira, R., Rodriguez-Diaz, J., Banerjee, S., Boutaud, A., Rubio, V., Hudson, B. G., Saus, J., Cervera, J., and Marina, A. (2018) Structures of collagen IV globular domains: insight into associated pathologies, folding and network assembly. *IUCrJ* **5**, 765-779

4. Wieslander, J., Langeveld, J., Butkowski, R., Jodlowski, M., Noelken, M., and Hudson, B. G. (1985) Physical and immunochemical studies of the globular domain of type IV collagen. Cryptic properties of the Goodpasture antigen. *J Biol Chem* **260**, 8564-8570

5. Borza, D. B., Bondar, O., Colon, S., Todd, P., Sado, Y., Neilson, E. G., and Hudson, B. G. (2005) Goodpasture autoantibodies unmask cryptic epitopes by selectively dissociating autoantigen complexes lacking structural reinforcement: novel mechanisms for immune privilege and autoimmune pathogenesis. *J Biol Chem* **280**, 27147-27154

6. Boutaud, A., Borza, D. B., Bondar, O., Gunwar, S., Netzer, K. O., Singh, N., Ninomiya, Y., Sado, Y., Noelken, M. E., and Hudson, B. G. (2000) Type IV collagen of the glomerular basement membrane. Evidence that the chain specificity of network assembly is encoded by the noncollagenous NC1 domains. *J Biol Chem* **275**, 30716-30724

7. Mizuno, K., Boudko, S. P., Engel, J., and Bachinger, H. P. (2010) Kinetic hysteresis in collagen folding. *Biophys J* **98**, 3004-3014

8. Pedchenko, V., Bondar, O., Fogo, A. B., Vanacore, R., Voziyan, P., Kitching, A. R., Wieslander, J., Kashtan, C., Borza, D. B., Neilson, E. G., Wilson, C. B., and Hudson, B. G. (2010) Molecular architecture of the Goodpasture autoantigen in anti-GBM nephritis. *N Engl J Med* **363**, 343-354

9. Cui, Z., Zhao, M. H., Jia, X. Y., Wang, M., Hu, S. Y., Wang, S. X., Yu, F., Brown, K. L., Hudson, B. G., and Pedchenko, V. (2016) Antibodies to alpha5 chain of collagen IV are pathogenic in Goodpasture's disease. *J Autoimmun* **70**, 1-11
